# Clinical outcomes of effectiveness and safety in cataract patients implanted with the novel hybrid extended depth-of-focus intraocular lens AM2UH: a prospective study

**DOI:** 10.3389/fmed.2026.1804708

**Published:** 2026-04-09

**Authors:** Qi Guo, Yuanjun Huang, Yinping Yang, Lu Yuan, Changyan Liu, Chunhong Xu, Wei Liu, Jilin Tan

**Affiliations:** 1 Chongqing Aier Eye Hospital, Aier Eye Hospital Group, Chongqing, China; 2 Department of Ophthalmology, The First School of Clinical Medicine, Jinan University, Guangzhou, Guangdong, China

**Keywords:** defocus curve, extend depth of focus, intraocular lens, photic phenomena, visual acuity

## Abstract

**Background:**

AM2UH was a novel bifocal hybrid extend the depth of focus (EDF) intraocular lens (IOL). This study is to evaluate the effectiveness and safety by comparing it with a monofocal IOL (AW-UV).

**Methods:**

A prospective, comparative, single-center study. 37 patients (37 eyes) were implanted with novel hybrid EDF IOL model AM2UH. 36 patients (36 eyes) were implanted with monofocal IOL model AW-UV. At 6 months postoperatively, the visual acuity (uncorrected and distance corrected), defocus curves, refractive outcomes, contrast sensitivity, questionnaires of visual quality were assessed and compared between 2 groups.

**Results:**

Compared to AW-UV, AM2UH showed non-inferior monocular corrected distance visual acuity (*p* > 0.05), but superior intermediate and near visual acuity (all *p* < 0.05). Additionally, AM2UH provided satisfactory DCVA at 1.0 m (0.15 ± 0.07 logMAR) and an extended depth of focus, with an increase of 1.08 D at the 0.2 logMAR. AM2UH group showed high spectacle independence rate and satisfaction rates about intermediate and near vision. There were no significant differences in contrast sensitivity, the incidences of photic phenomena, intraocular pressure and adverse events between 2 groups.

**Conclusion:**

AM2UH IOL is an effective and safe EDF IOL that offers satisfactory intermediate vision and distance vision, improves near vision, and delivers an extended visual range while maintaining favorable visual quality.

**Clinical trial registration:**

Identifier: MR-50-25-086616.

## 1 Introduction

In recent years, driven by the growing demand for higher visual quality among the general public, cataract surgery has progressively transitioned from a blindness-preventing procedure into the era of refractive cataract surgery (1, 2). While traditional monofocal and bifocal intraocular lenses (IOLs) can provide good distance vision and distance/near vision, respectively, they often fail to meet the intermediate vision needs of cataract patients (3–5). Extended depth of focus (EDF) technology addresses this by distributing light to a more extended focal point, thereby achieving an elongated focus (6–8). Various optical designs can achieve the EDF effect. For instance, the Tecnis Symfony (Johnson & Johnson Vision, Inc. United States) utilizes a diffractive design (9), the AcrySof IQ Vivity (Alcon Laboratories, Inc. United States) employs a wavefront-shaping design (10), and the Tecnis PureSee (Johnson & Johnson Vision, Inc. United States) relies on a refractive design (11). All these IOLs have demonstrated effectiveness in improving intermediate vision and reducing dependence on glasses.

The AM2UH IOL is distinct from the aforementioned designs. It employs a high-order aspheric design on the anterior surface to introduce controlled higher-order aberrations, elongating the focal point and achieving an extended depth of focus of over 1.0 D. This is combined with a diffractive bifocal platform (with an add power of +2.4 D) on the posterior surface (12, 13). This innovative hybrid design aims to enhance intermediate vision and improve postoperative visual continuity and overall visual quality. Although *in vitro* MTF assessment studies have confirmed its capacity to provide a certain continuity of the visual range (under 3.0 mm and 4.5 mm pupil diameters, at a spatial frequency of 50 lp/mm), more direct clinical evidence has not yet been reported (12). The American National Standards Institute (ANSI) and the International Organization for Standardization (ISO) have established clear benchmarks for the clinical performance of EDF IOLs (14, 15). Therefore, the purpose of this study is to demonstrate the clinical effectiveness and safety of the AM2UH IOL as an EDF IOL by comparing with a monofocal IOL.

## 2 Methods

### 2.1 Study design

This was a prospective, comparative, single-center study after implantation of a novel hybrid EDF IOL model AM2UH (Eyebright Medical Technology (Beijing) Co., Ltd., China) or a monofocal IOL model AW-UV (Eyebright Medical Technology (Beijing) Co., Ltd., China), conducted in China between December 2024 and August 2025. The study adhered to the principles of the Declaration of Helsinki and was approved by the ethics committees of Chongqing Aier Eye Hospital (Approval No.: 202412019). Written informed consent was obtained from all patients. This study was formally registered in the official Chinese “Medical Research Registration and Filing Information System” (Registration No. [MR-50-25-086616]).

### 2.2 Patients

The sample size was calculated using PASS software (version 2021, NCSS, LLC). The primary variable used for the calculation was monocular best-corrected distance visual acuity under photopic conditions. A non-inferiority trial design was adopted, with the non-inferiority margin (*δ*) set at 0.1 logMAR (16). Assuming a standard deviation (*σ*) of 0.1 logMAR for both groups, and using a one-sided significance level of *α* = 0.05 (corresponding to a 90% confidence interval) and a power (1 − *β*) of 80%, the minimum required sample size was 13 eyes per group. Ultimately, 37 and 36 participants were enrolled in the AM2UH group and AW-UV group, respectively, meeting and exceeding the required sample size.

We included patients who were aged 18 years or older and had cataract with in at least one eye. Exclusion criteria included ocular comorbidities including (myopic macular degeneration, glaucoma, and so on), uncontrolled elevated intraocular pressure (IOP), a potential postoperative monocular corrected distance visual acuity (CDVA) anticipated to be ≤0.5 decimal acuity (e.g., foveal abnormalities); or the use of medications that could affect visual acuity.

### 2.3 Assessments

All eyes in the study were targeted to emmetropia, AM2UH targets the minimum absolute residual error (closest to 0.00 D); AW-UV targets the smallest negative residual error (closest to 0.00 D from the minus side) (17). Six scheduled study visits were planned for each patient: screening, the operative visit, and postoperative visits at days 1 to 2, 1 week, 1 month, and 6 months.

Under 100% contrast, distance, intermediate and near visual acuity assessment was conducted monocularly at 5 m, 66 cm, and 40 cm, respectively, including uncorrected distance visual acuity (UDVA), corrected distance visual acuity (CDVA), uncorrected intermediate visual acuity (UIVA), distance corrected intermediate visual acuity (DCIVA) and uncorrected near visual acuity (UNVA) and distance corrected near visual acuity (DCNVA). At 10% contrast, intermediate visual acuity assessment was also conducted monocularly at 66 cm, including UIVA, DCIVA and best corrected intermediate visual acuity (BCIVA). All visual acuity assessment were conducted using logMAR Chart. Defocus curves were assessed under best-corrected distance vision using trial lenses from +1.50 D to −3.50 D. The defocus was introduced in 0.50 D steps, with a finer step of 0.25 D between +0.50 D and −0.50 D, to evaluate visual acuity. The depth of focus was determined by using data from all subjects of each group. Contrast sensitivity was tested in mesopic conditions (with and without glare) using a CSV-1000 E contrast chart. IOP was measured using a non-contact tonometer. The Chinese version of the Visual Function Questionnaire-14 (VF-14-CN) (18–20) was administered to evaluate postoperative visual function and quality of life. Additionally, the rate of visual disturbances, satisfaction and spectacle dependence for distance, intermediate, and near vision were measured using a validated IOL satisfaction questionnaire (21).

### 2.4 End points and outcomes

The 4 coprimary effectiveness objectives of the study, developed in line with ISO EDF IOL criteria (15), were to demonstrate that at 6 months postoperatively: (1) AM2UH was noninferior to AW-UV regarding monocular CDVA, using a noninferiority margin of 0.10 logMAR; (2) monocular mean DCIVA of AM2UH was 0.2 logMAR or better, and superior to AW-UV; (3) the monocular mean defocus curve for AM2UH had a negative range at least 0.5 D greater than AW-UV at 0.2 logMAR; and (4) the distance corrected visual acuity (DCVA) at 1.0 m of AM2UH was 0.2 logMAR or better.

Additional effectiveness end points included the visual acuity (distance, intermediate and near), spherical equivalent (SE) and the mesopic contrast sensitivity. The contrast sensitivity was assessed at 1.5, 3, 6, and 12 cycles per degree (with and without glare) at 6 months postoperatively. The results of VF-14-CN and IOL satisfaction questionnaire at 6 months postoperatively were also served as the effectiveness end points.

The 2 coprimary safety end points were the IOP and the rate of adverse events.

### 2.5 Intraocular lenses

**Figure 1:**
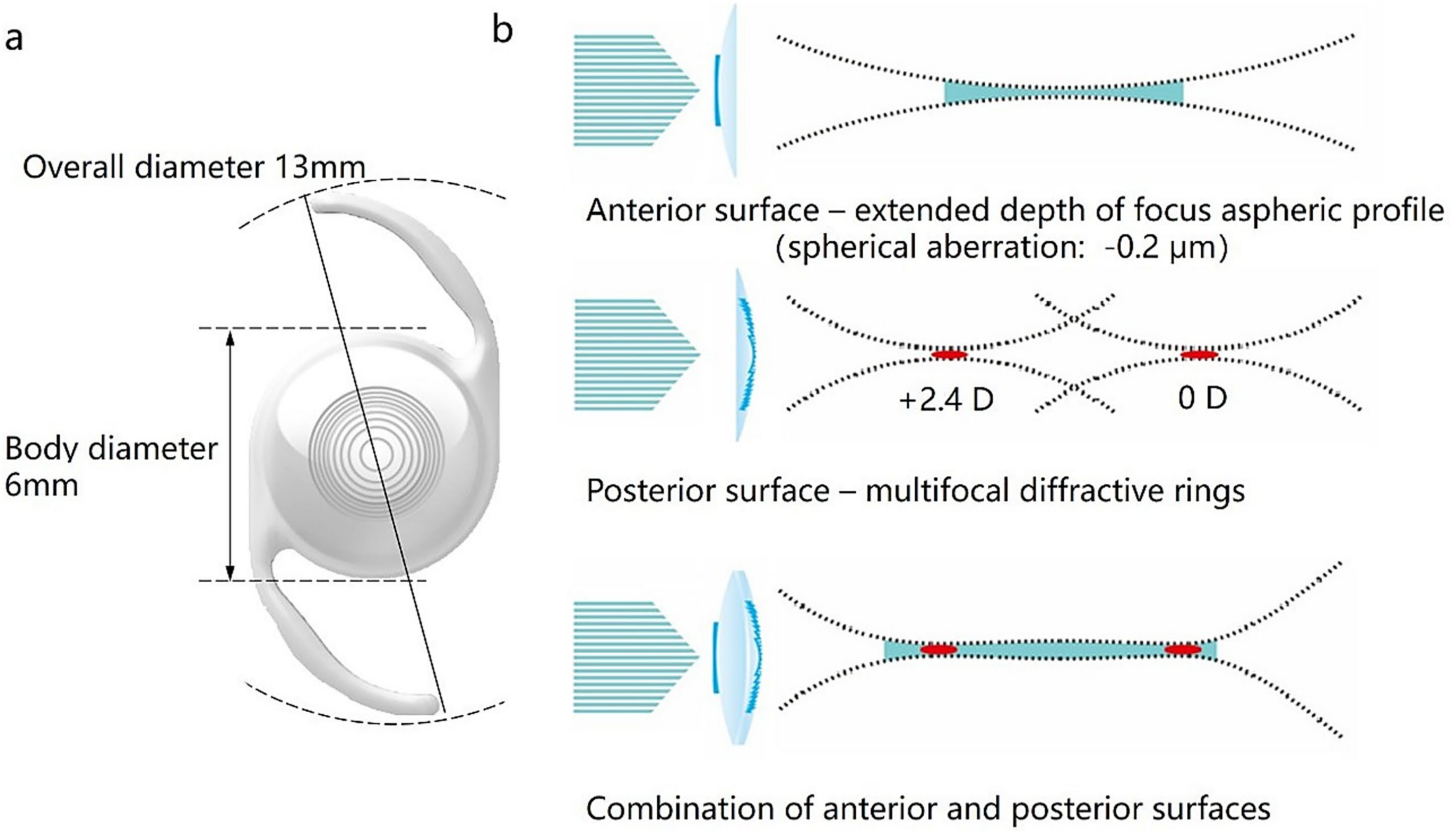
**(a)** Front view of AM2UH. **(b)** Illustration showing the mechanism of action of AM2UH.

The novel EDF IOL (model: AM2UH) is a one-piece/posterior chamber intraocular lens featuring a 6.0 mm aspheric optic with an overall diameter of 13 mm (Figure 1a). This intraocular lens features an innovative synergistic optical design utilizing both the anterior and posterior surfaces: the posterior surface employs a diffractive bifocal design with an added power of +2.4 D to direct light to both distant and near focal points, thereby providing distance and intermediate/near vision; the anterior surface utilizes a higher-order aspheric design to introduce controlled higher-order aberrations, elongating the focal point and extending the depth of focus(Figure 1b). This achieves an extended depth of focus of over 1.0 D, which, combined with the eye’s inherent physiological depth of focus, optimizes intermediate vision and collectively expands the range of clear vision, resulting in continuous vision. The available lens power range is from +6.0 D to +30.0 D in 0.5 D increments.

Monofocal IOL (Model AW-UV) is a one-piece hydrophobic aspheric intraocular lens with a 6.0 mm aspheric optic and an overall diameter of 13 mm. The lens power ranges from −10.0 D to +36.0 D in 0.5 D increments.

### 2.6 Statistical analysis

SPSS (version 22.0) was used for analysis. The normality of data distribution was assessed using the Shapiro–Wilk test. The mean and standard deviation were used as descriptive statistics. Quantitative data were assessed using the Independent Samples *t*-test and the Mann–Whitney *U* test as suitable. Categorical variables are expressed as frequency (*n*, %) and were compared using the *χ*^2^ test. *p*-value of less than 0.05 was considered statistically significant.

## 3 Results

### 3.1 Baseline characteristics

**Figure 2:**
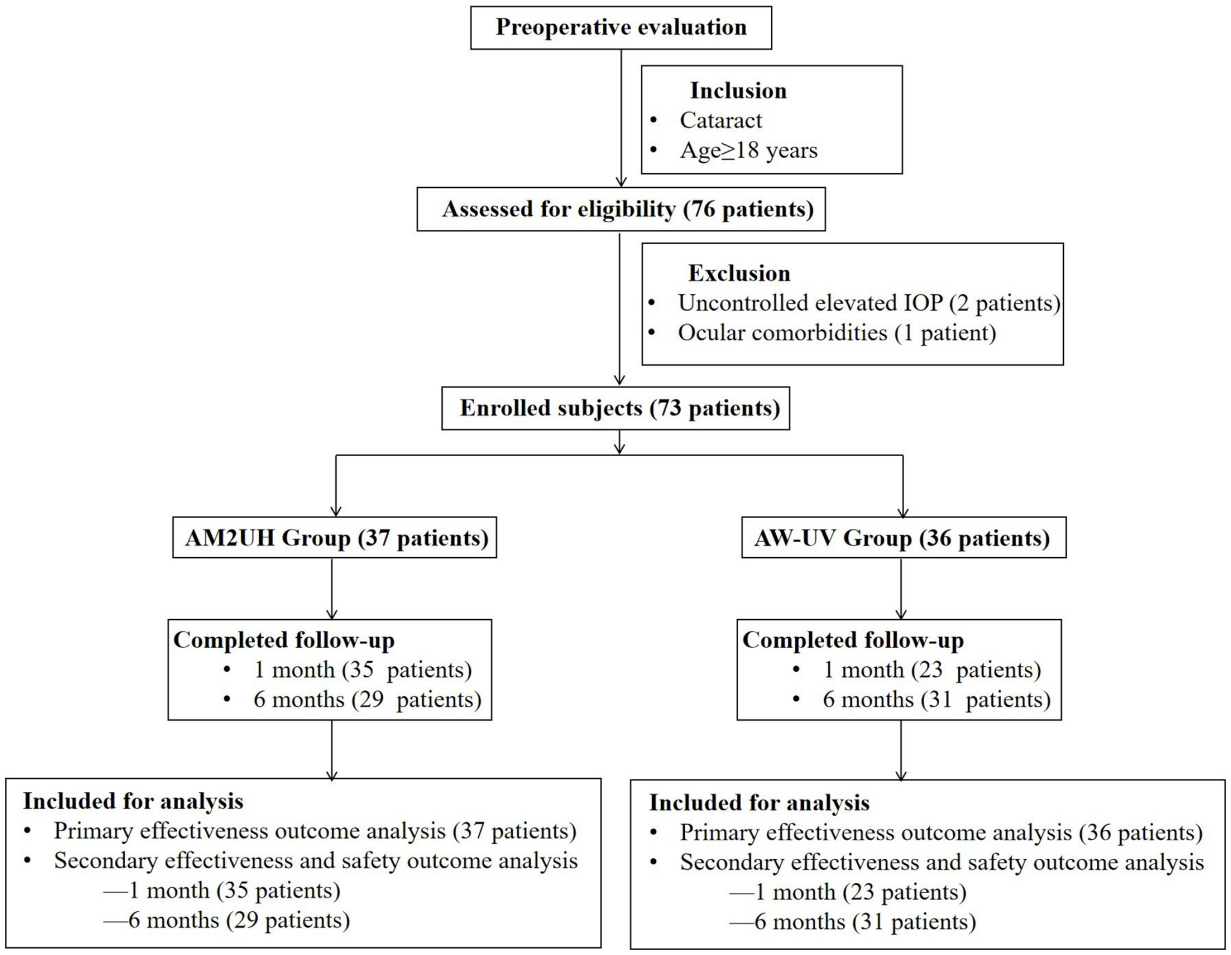
Enrollment and follow-up flow chart.

A total of 73 patients were enrolled: 37 received the novel hybrid EDF IOL model AM2UH and 36 received the monofocal IOL model AW-UV (Figure 2). The mean age was 68.8 ± 8.9 years (range: 41 to 85 years). There were no statistically significant differences between the two groups with regard to patient demographics or baseline characteristics (Table 1). All surgical procedures were uneventful and all IOLs were implanted into the capsular bag.

**Table 1:** Patient demographics and baseline characteristics.

Parameter	AM2UH (*n* = 37)	AW-UV (*n* = 36)	*p*
Sex			
Female	22 (59.5%)	25 (69.4%)	0.373
Male	15 (40.5%)	11(30.6%)	
UDVA (logMAR)	0.75 ± 0.45	0.88 ± 0.47	0.283
ACD (mm)	3.12 ± 0.39	3.02 ± 0.48	0.356
AL (mm)	23.32 ± 0.80	23.43 ± 0.94	0.608
SE (D)	−0.80 ± 2.71	−0.62 ± 2.65	0.911
IOP (mmHg)	14.03 ± 3.32	13.86 ± 2.96	0.820
IOL power (D)	20.58 ± 2.53	20.61 ± 2.59	0.959

Except for sex, values are presented as mean ± standard deviation (SD). *n*, number of subjects; UDVA, uncorrected distance visual acuity; CDVA, corrected distance visual acuity; ACD, anterior chamber depth; AL, axial length; SE, spherical equivalent; IOP, intraocular pressure.

### 3.2 Coprimary effectiveness outcomes

At 6 months postoperatively, the 4 coprimary effectiveness outcomes are presented in Table 2. AM2UH provided noninferior CDVA compared with AW-UV (0.03 ± 0.05 logMAR vs. 0.06 ± 0.07 logMAR, *p* > 0.05), and the upper limit of the 90% confidence interval (CI) of the mean difference between 2 groups was 0.01 logMAR, less than 0.1 logMAR (noninferiority margin). AM2UH provided superior better DCIVA compared with AW-UV (0.13 ± 0.11 logMAR vs. 0.28 ± 0.17 logMAR, *p* < 0.05), with upper limit of the 95% CI was −0.08 logMAR, less than 0 (superiority margin). Meanwhile, AM2UH provided a 1.09 D (≥0.5 D) wider negative defocus range at 0.20 logMAR than AW-UV group, with a DCVA of 0.15 logMAR at 1.0 m.

**Table 2:** Coprimary effectiveness outcomes at 6 months postoperatively.

Parameter	AM2UH	AW-UV	Between-group difference		Margin	*p*-value
	Mean (±SD)	Mean (±SD)	Mean (±SD)	90% or 95% CI		
CDVA (logMAR)	0.03 ± 0.05	0.06 ± 0.07	−0.03 ± 0.04	(−0.06, 0.01)	0.1	0.104
DCIVA (logMAR)	0.13 ± 0.11	0.28 ± 0.17	−0.15 ± 0.04	(−0.23, −0.08)	0	<0.001
Depth of focus at 0.2 logMAR (D)	2.00 ± 0.61	0.92 ± 0.53	1.08			
DCVA at 1.0 m (logMAR)	0.15 ± 0.07	0.24 ± 0.11				<0.001

Values are presented as mean ± standard deviation. CDVA, corrected distance visual acuity; DCIVA, distance corrected intermediate visual acuity; D, diopter; DCVA, distance corrected visual acuity; CI, confidence interval; CDVA, non-inferiority margin was 0.1 logMAR, 90% CI; DCIVA: superiority margin was 0.1 logMAR, 95% CI.

### 3.3 Visual acuity

**Figure 3:**
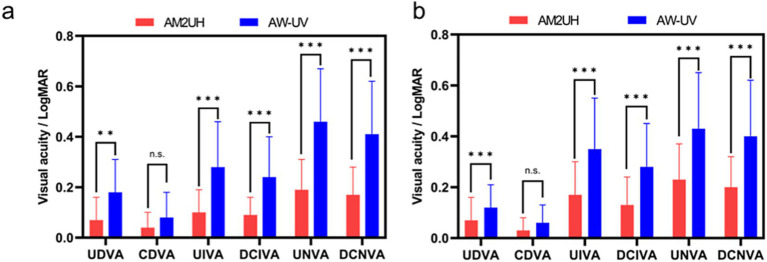
Visual acuity at 1 month **(a)** and 6 months **(b)** after surgery for eyes implanted with AM2UH vs. AW-UV at 100% contrast. Differences between the AM2UH and AW-UV groups are denoted as: *** for *p* < 0.001, ** for *p* < 0.01, * for *p* < 0.05, and n.s. for not significant (*p* > 0.05).

**Figure 4:**
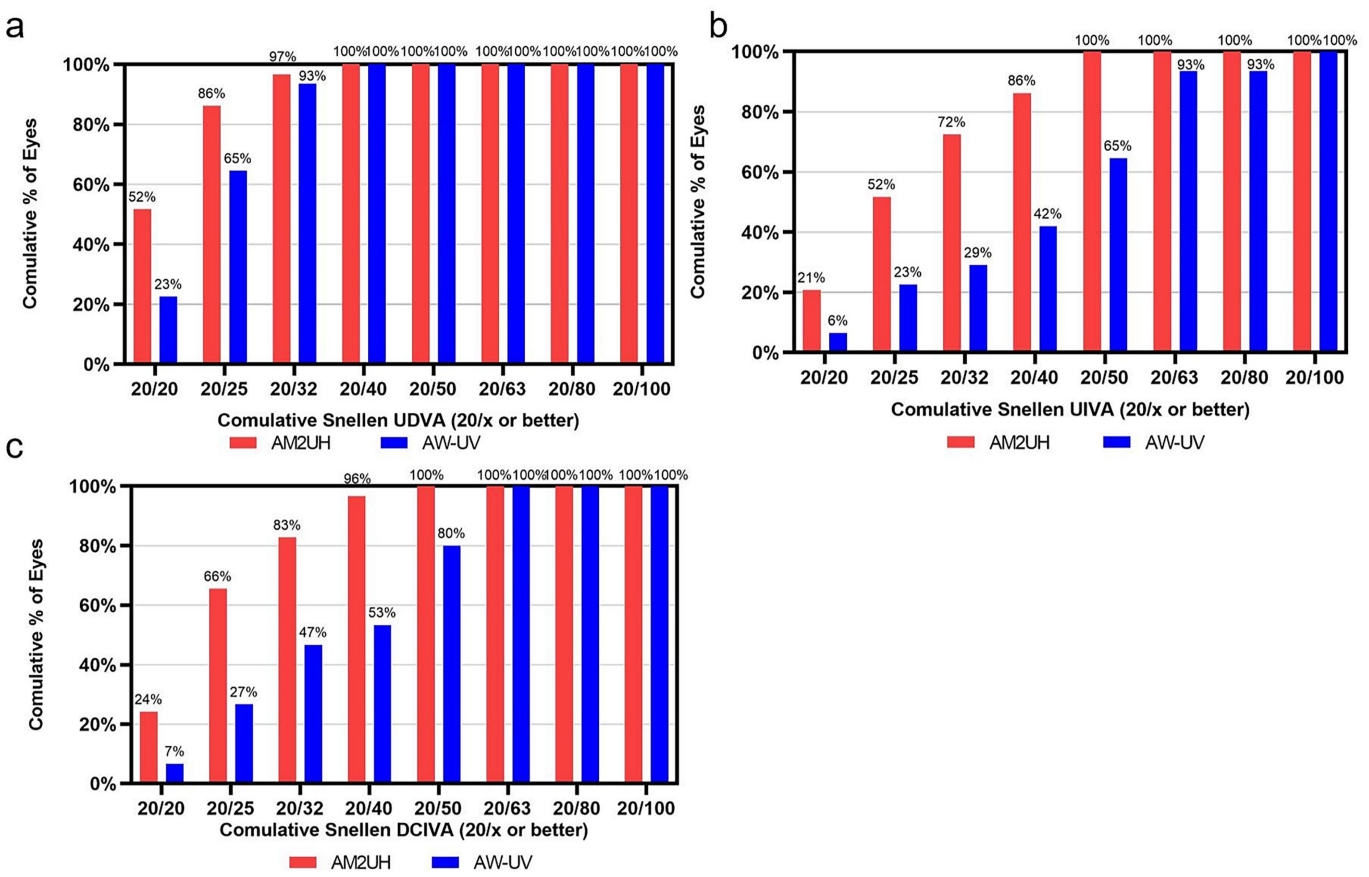
Cumulative distribution of 6-months postoperative visual acuity for eyes implanted with AM2UH vs. AW-UV at 100% contrast: **(a)** UDVA; **(b)** UIVA; **(c)** DCIVA. n.s. for not significant (*p* > 0.05).

At 100% contrast, the visual acuity outcomes for the AM2UH and AW-UV groups are presented in Figure 3. The AM2UH group demonstrated greater intermediate (UIVA and DCIVA), near visual acuity (UNVA and DCNVA) and UDVA compared to the AW-UV group (all *p* < 0.05), while no statistically significant difference was found in CDVA (*p* > 0.05). The proportion of patients achieving 20/20 Snellen or better was higher in the AM2UH group than the AW-UV group for UDVA (51.7% vs. 22.6%). Similarly, the AM2UH group demonstrated higher rates of achieving 20/32 Snellen or better in UIVA (72.4% vs. 29.3%) and DCIVA (82.7% vs. 46.7%) (Figure 4).

At 10% contrast, the AM2UH group demonstrated greater intermediate (UIVA, BCIVA, DCIVA) visual acuity (*p* < 0.05) (Table 3).

**Table 3:** Visual acuity (logMAR) at 10% contrast, 6 months postoperatively.

Parameter	AM2UH	AW-UV	*p*
UIVA	0.51 ± 0.16	0.61 ± 0.15	0.012
BCIVA	0.49 ± 0.14	0.61 ± 0.15	0.004
DCIVA	0.48 ± 0.14	0.61 ± 0.15	0.002

Values are presented as mean ± standard deviation. UIVA, uncorrected intermediate visual acuity; DCIVA, distance corrected intermediate visual acuity; BCIVA, best corrected intermediate visual acuity.

### 3.4 Refractive outcomes

**Figure 5:**
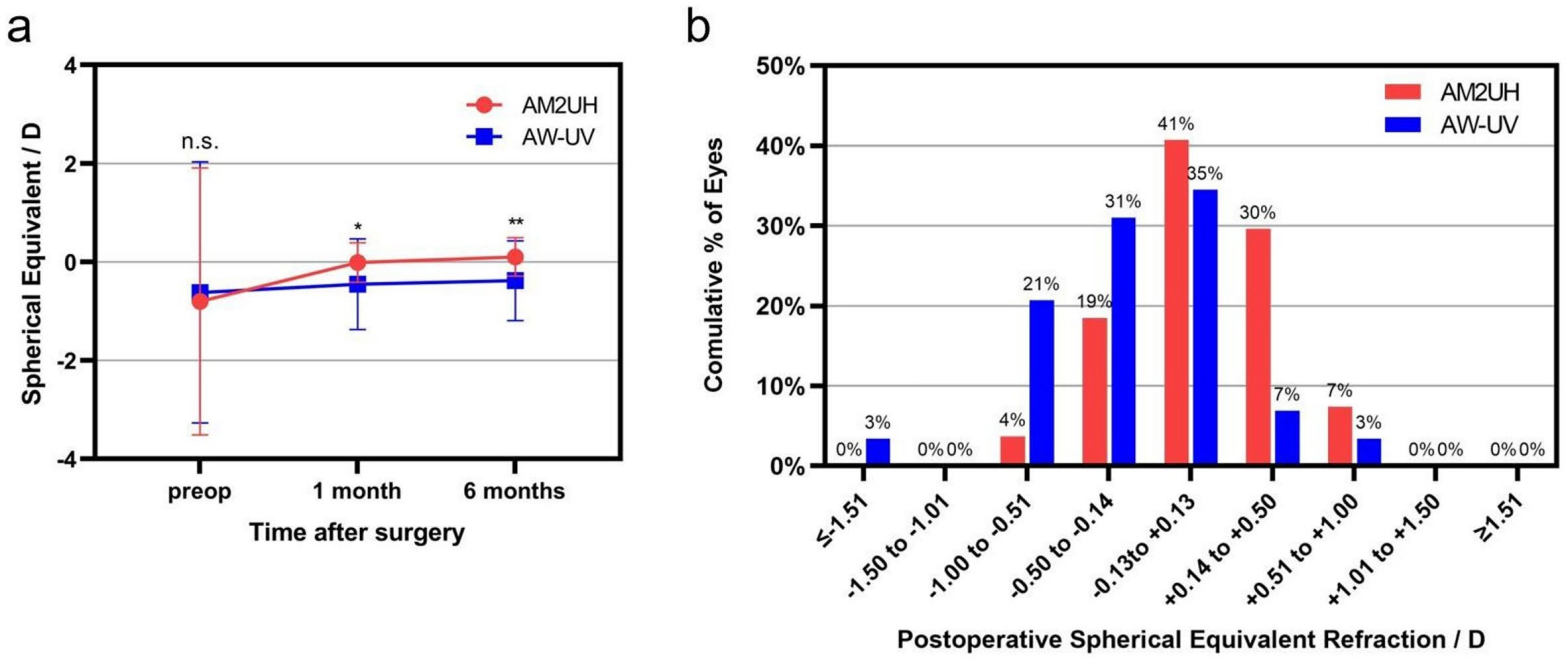
**(a)** The comparison of spherical equivalent (SE) at different time. **(b)** The distribution of the SE at 6 months postoperatively. Differences between the AM2UH and AW-UV groups are denoted as: ** for *p* < 0.01, * for *p* < 0.05, and n.s. for not significant (*p* > 0.05).

At 6 months postoperatively, the mean SE in the AM2UH group (0.10 ± 0.40 D) approximated emmetropia more closely than that in the AW-UV group (−0.38 ± 0.81 D) (*p* < 0.05) (Figure 5a). At 6 months postoperatively, 88.8% of eyes in the AM2UH group and 72.4% of eyes in the AW-UV group were within 0.50 D of target refraction (Figure 5b).

### 3.5 Defocus curve

**Figure 6:**
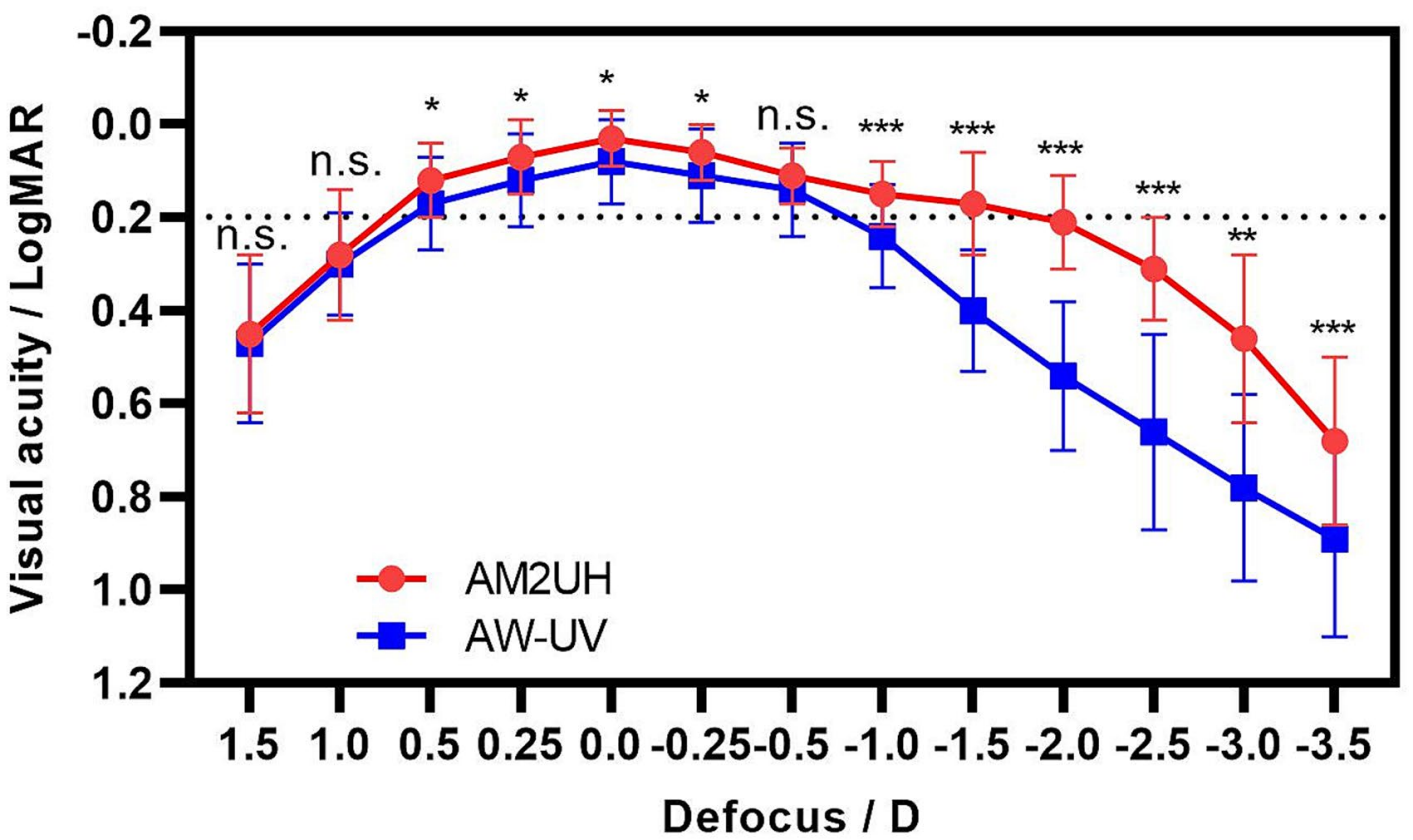
The defocus curves of AM2UH and AW-UV. Differences between the AM2UH and AW-UV groups are denoted as: *** for *p* < 0.001, ** for *p* < 0.01, * for *p* < 0.05, and n.s. for not significant (*p* > 0.05).

At 6 months postoperatively, both defocus curves showed a peak at a defocus 0 D (Figure 6). The AM2UH exhibited a flatter curve, in contrast to the AW-UV, which showed abrupt declines from 0 D. From −1.0 D (1.0 m) to −3.5 D (28 cm), AM2UH achieved statistically significantly better visual acuity than the AW-UV (all *p* < 0.01), indicating its superior performance for both intermediate and near vision. These findings are in agreement with the measurements of DCIVA and DCNVA. Compared to AW-UV, AM2UH demonstrated comparable distance visual acuity. The depth of focus was 1.08 D wider in the AM2UH (novel hybrid EDF IOL) group than in the AW-UV (monofocal IOL) group at 0.2 logMAR (2.00 D vs. 0.92 D).

### 3.6 Contrast sensitivity

**Figure 7:**
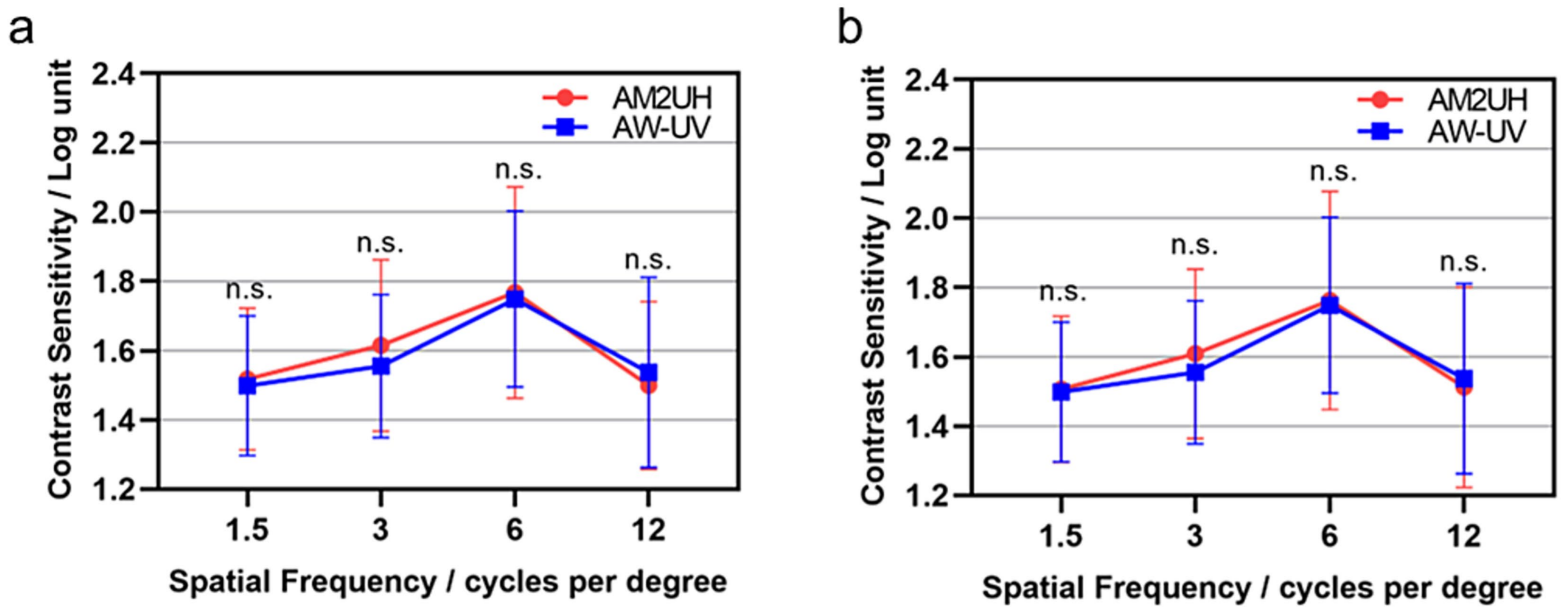
Mean contrast sensitivity in mesopic condition without glare **(a)** and with glare **(b)** at 6 months postoperatively. n.s., no statistically significant difference between the AM2UH and AW-UV groups (*p* > 0.05).

At 6 months postoperatively, under mesopic conditions, the AM2UH IOL showed no statistically significant difference in contrast sensitivity compared to the AW-UV group, both with and without glare (Figures 7a,b).

### 3.7 Patient questionnaires

**Figure 8:**
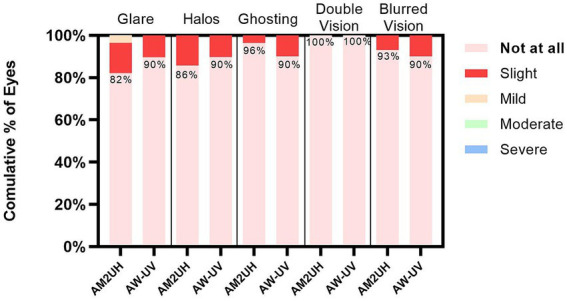
Incidence of visual disturbances at 6 months postoperatively.

At 6 months postoperatively, the majority of patients in both the AM2UH and AW-UV groups remained free from photic phenomena (*p* > 0.05) (Figure 8). Compared to the AW-UV group, the AM2UH group demonstrated higher satisfaction rates with intermediate and near vision, as well as higher rates of spectacle independence at intermediate and near distances (all *p* < 0.001) (Table 4).

**Table 4:** The results of questionnaire regarding satisfaction, spectacle dependence, and VF-14-CN scores.

Parameter	AM2UH	AW-UV	*p*
Satisfaction			
Distance	100%	96.7%	0.492
Intermediate	100%	69.0%	<0.001
Near	89.3%	41.4%	<0.001
Spectacle independence			
Distance	100%	96.7%	1.000
Intermediate	100%	43.3%	0.001
Near	85.7%	30.0%	<0.001
VF-14-CN			
Total score, mean ± SD	97.11 ± 5.5	89.93 ± 9.65	0.032

VF-14-CN, visual function questionnaire (Chinese version); SD, standard deviation.

### 3.8 Intraocular pressure

**Figure 9:**
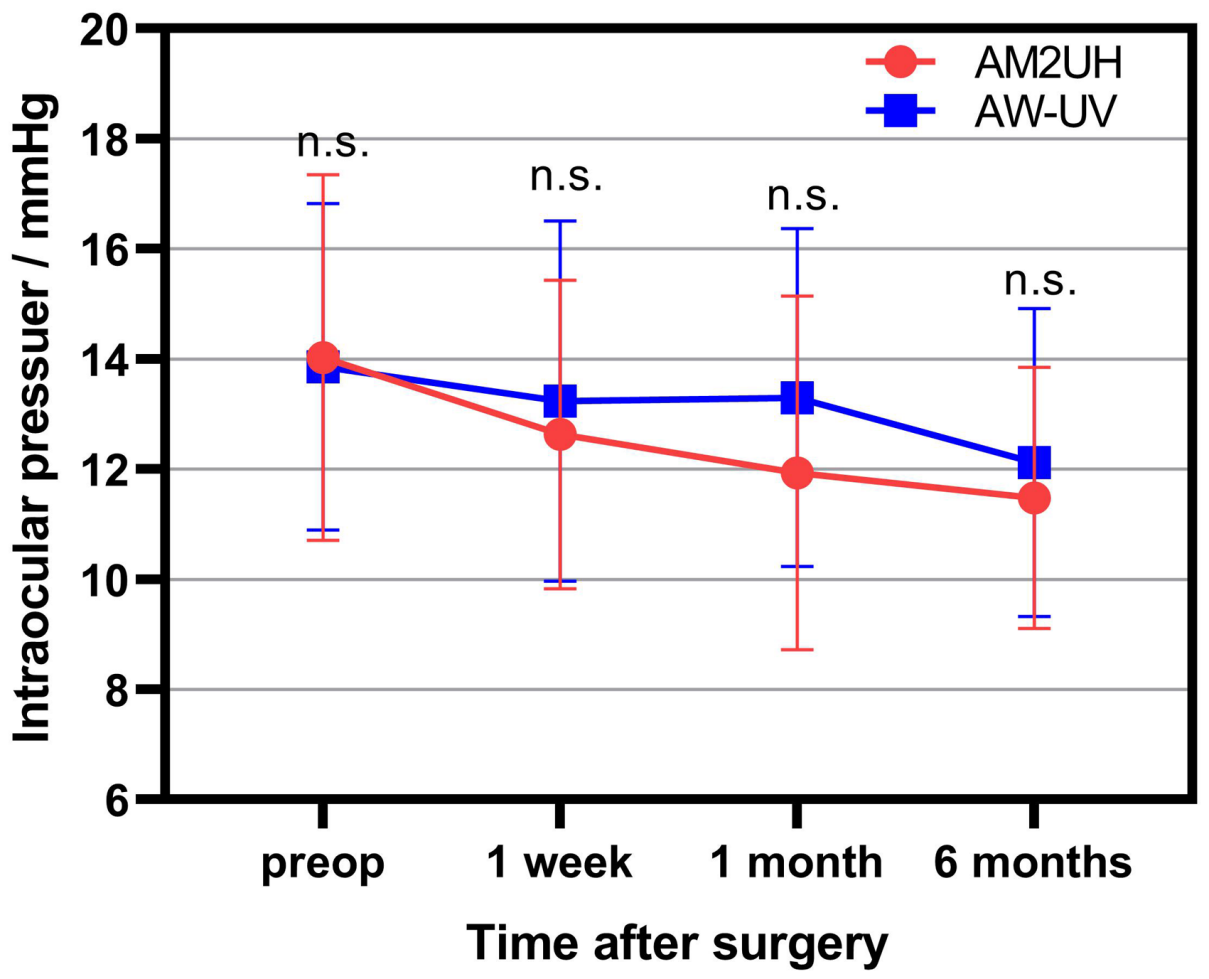
The intraocular pressure of the AM2UH group and the AW-UV group. n.s., no statistically significant difference between AM2UH and AW-UV groups (*p* > 0.05).

From preoperative to 6 months postoperative, there was no statistically significant difference in the mean IOP between the AM2UH and AW-UV groups (Figure 9).

### 3.9 Adverse events

Postoperative complications and adverse events in the AM2UH group (EDF IOL) were comparable to those observed with AW-UV (monofocal IOL). The incidence rates in both groups fell below the safety and performance endpoints (SPE) rate in ISO11979-7:2024 (15) (Table 5), demonstrating favorable clinical safety of the AM2UH.

**Table 5:** Comparison of adverse event rate and SPE rate.

Parameter	This study				ISO criteria (15)		
Adverse event	AM2UH (37 cases)		AW-UV (36 cases)		SPE rate (%)	Threshold rate (%)	Max. *N* allowed before SPE rate exceeded (100 cases)
	*N*	Rate (%)	*N*	Rate (%)			
Cumulative							
Cystoid macular oedema	1	2.7	1	2.8	3.0	8.9	6
Hypopyon	0	0.0	0	0.0	0.3	3.0	1
Endophthalmitis	0	0.0	0	0.0	0.1	3.0	1
Lens dislocated from posterior chamber	0	0.0	0	0.0	0.1	3.0	1
Pupillary block	0	0.0	0	0.0	0.1	3.0	1
Retinal detachment	0	0.0	0	0.0	0.3	3.0	1
Secondary surgical intervention	0	0.0	0	0.0	0.8	4.2	2
Persistent							
Corneal stroma oedema	0	0.0	0	0.0	0.3	3.0	1
Cystoid macular oedema	0	0.0	0	0.0	0.5	4.2	2
Iritis	0	0.0	0	0.0	0.3	3.0	1
Raised IOP requiring treatment	0	0.0	0	0.0	0.4	4.2	2

*N*, Number of cases; SPE, safety and performance endpoints. SPE rates were derived from weighted averages of the data from large clinical investigations of anterior and posterior chamber IOLs (15).

## 4 Discussion

The growing demand for optimal vision during intermediate tasks, such as computer use, while preserving distance vision quality led to the development of EDF IOLs (22). With the continuous updates of EDF IOLs, international consensus and standards for EDF IOLs are also being successively issued and updated (14, 15, 23). Most recently, ISO 11979-7:2024 has clearly defined the clinical outcomes of EDF IOLs, Including CDVA, DCIVA, depth of focus, incidence of adverse events, and so on. Therefore, this clinical trial was conducted to demonstrate that the AM2UH, as a novel bifocal hybrid EDF IOL, meets the clinical effectiveness and safety requirements specified by ISO.

In this prospective, comparative, single-center study, the AM2UH IOL met the ISO 11979-7:2024 (15) criteria for EDF IOLs by providing a significantly improved range of vision from distance to near compared to the AW-UV monofocal IOL, without increasing the incidence of visual disturbances.

Comparing with monofocal IOL, AM2UH provided superior DCIVA and non-inferior CDVA at 6 months postoperatively. This result is consistent with the study of other EDF IOL model DFT015 (AcrySof IQ Vivity) (24).

In terms of postoperative visual acuity, this study demonstrated that the AM2UH successfully extended the range of vision from distance to near, with significantly enhanced intermediate and near visual performance compared to the monofocal IOL, while maintaining comparable and excellent distance visual acuity. Postoperative UDVA was slightly better in the AM2UH group than in the AW-UV group (0.07 ± 0.09 logMAR vs. 0.12 ± 0.09 logMAR, *p* = 0.013), which is consistent with the SE results indicating that AM2UH approximated emmetropia more closely (0.10 ± 0.39 D vs. −0.38 ± 0.81 D, *p* < 0.001). Although there was significantly difference in mean SE between 2 groups, both the AM2UH and AW-UV IOLs demonstrated comparable and favorable predictability, with 88.8% and 72.4% of eyes achieving a spherical equivalent (SE) within ±0.50 D of the target, respectively (*p* > 0.05). More importantly, when assessed DCIVA and DCNVA, which eliminate the influence of residual refraction, the AM2UH group still demonstrated significantly better visual acuity. This confirms that the EDF design of the AM2UH, rather than the slight difference in postoperative refraction, is the primary driver for its enhanced visual performance across distances.

Diffractive bifocal IOLs provide somewhat poor intermediate visual acuity (25). However, in our study, AM2UH achieved better monocular outcomes at both 1 and 6 months (0.10 ± 0.09 and 0.18 ± 0.13 logMAR, respectively) for UIVA at 66 cm, consistent with the UIVA of Tecnis Eyhance at 6 months (0.18 ± 0.07 logMAR) (26).

According to the literature review by Srinivasan et al. (27), compared to monofocal IOLs (549 eyes), the defocus curve of EDF IOLs (1898 eyes) demonstrated a significant improvement in visual acuity for the intermediate to near range (−1.5 D to −2.0 D). In this study, the defocus curve of AM2UH showed significantly better visual acuity from −1 D to −3 D, which is consistent with the findings in the literature review (27). Additionally, the depth of focus of the monofocal IOLs in this study was 0.92 D, aligning with the 1.17 D reported for monofocal IOLs in the review (27). Besides, the defocus curve of AM2UH exhibits a plateau of visual acuity (0.2 logMAR or better) from +0.5 D to −2.0 D, which differs from the single-peak (0 D) of monofocal IOLs. Furthermore, the visual acuity at 1.0 m (−1.0 D on the defocus curve) of AM2UH was 0.15 logMAR, meeting the ISO criteria of being less than 0.2 logMAR. Berdahl et al. (28) reported similar results for the Clareon Vivity IOL (Alcon Laboratories, Inc., United States): a depth of focus of 1.7 D and a visual acuity of approximately 0.15 logMAR at 1.0 D. Therefore, compared to the current clinically dominant EDOF IOLs [e.g., Symfony ZXR00 1.75 D (29), PureSee ZEN00V 1.7 D (30, 31)], AM2UH provides a comparable depth of focus.

Contrast sensitivity is a key outcome measure following IOL implantation, reflecting the visual system’s ability to distinguish differences in luminance of static images (32). According to ISO 11979-7:2024, the mean differences of contrast sensitivity between EDF IOLs and monofocal IOLs should be less than 0.3 log units at any frequency. This study demonstrated that the mean difference in contrast sensitivity between the AM2UH and AW-UV was within 0.1 log units and was not statistically significant (all *p* > 0.05). This indicates that the AM2UH provides an extended range of vision without compromising contrast sensitivity, meeting the ISO criteria. The excellent contrast sensitivity achieved with the AM2UH IOL may be attributed to its aspheric optical design featuring −0.2 μm spherical aberration.

Subjective benefits and spectacle independence over time are significant factors driving patients’ expectations (33, 34). At the 6 months postoperatively, patients in the AM2UH group reported significantly higher rates of spectacle independence than those in the monofocal IOL group. Specifically, 100% of subjects in the AM2UH group did not wear spectacles for intermediate tasks (compared to 69%), and 89.3% for near tasks (compared to 41.1%). A meta-analysis of multifocal IOLs found that approximately 80% of patients achieved spectacle independence, highlighting that the AM2UH, as a hybrid EDF IOL, outperformed traditional multifocal IOLs in achieving spectacle independence (35).

Meanwhile, patient satisfaction rates with the AM2UH were significantly higher for both intermediate (100% vs. 43.3%) and near vision (41.4% vs. 30%) than with the monofocal IOL. These results further confirmed the AM2UH’s effectiveness in enhancing intermediate and near visual acuity postoperatively, aligning with findings from prior studies of EDF IOL (36).

Optical phenomena are a concern with any IOL (37). In our study, 18%, 14%, 4%, 0%, and 7% patients in the AM2UH group reported glare, halos, ghosting, double vision, and blurred vision, respectively. The corresponding numbers for the AW-UV group were 10%, 10%, 0%, 0%, and 10%. Intergroup comparison showed no statistically significant difference in the incidence rates. This indicates that the high-order aspheric and diffractive multifocal design does not increase the incidence of postoperative adverse visual phenomena, whereas previous studies on diffractive EDF IOLs (such as the TECNIS Symfony) reported a high rate of visual disturbances (36%) (38). The introduction of high-order aspheric EDF enhances the continuity between the two focal points of traditional diffractive lenses, thereby providing superior subjective visual perception compared to Symfony, which solely employs diffractive technology (39, 40).

The safety profile, including postoperative IOP, ocular health was comparable between the AM2UH and AW-UV groups. Notably, the incidence of complications and adverse events was not significantly different between 2 groups and fell below the SPE rate in ISO11979-7:2024. Those demonstrated the safety of AM2UH implantation.

This study has several limitations. First, the sample size was small and it was a single-center study. Future research should involve multi-center studies with larger sample sizes to enhance the conclusiveness of the findings. Second, this study was non-randomized and there was a lack of blinding to IOL type, which may have led to subjective bias in the measurement outcomes and affected the validity of the results. Third, the postoperative follow-up duration was short; extending it to 12 months or longer would better demonstrate the long-term effectiveness of the AM2UH in improving visual outcomes.

## 5 Conclusion

In conclusion, this study demonstrated the effectiveness and safety of the AM2UH IOL, which complies with the ISO-11979-7:2024 criteria for EDF IOLs. The lens provides satisfactory intermediate and near vision, with distance vision that is non-inferior to a monofocal IOL. Furthermore, it delivers an extended range of continuous vision without sacrificing contrast sensitivity or overall visual quality. For patients aiming to achieve spectacle freedom at both intermediate and far distances, the AM2UH presents a compelling choice.

## Data Availability

The raw data supporting the conclusions of this article will be made available by the authors, without undue reservation.
